# Parkinson’s disease and Alzheimer’s disease: a Mendelian randomization study

**DOI:** 10.1186/s12881-018-0721-7

**Published:** 2018-12-31

**Authors:** Zhifa Han, Rui Tian, Peng Ren, Wenyang Zhou, Pingping Wang, Meng Luo, Shuilin Jin, Qinghua Jiang

**Affiliations:** 1 School of Life Science and Technology, Harbin Institute of Technology, Harbin, China; 2 Department of Mathematics, Harbin Institute of Technology, Harbin, China

**Keywords:** Alzheimer’s disease, Parkinson’s disease, α-synuclein, Mendelian randomization, Genetic association

## Abstract

**Background:**

Alzheimer’s disease (AD) and Parkinson’s disease (PD) are the top two common neurodegenerative diseases in elderly. Recent studies found the α-synuclein have a key role in AD. Although many clinical and pathological features between AD and PD are shared, the genetic association between them remains unclear, especially whether α-synuclein in PD genetically alters AD risk.

**Results:**

We did not obtain any significant result (OR = 0.918, 95% CI: 0.782–1.076, *P* = 0.291) in MR analysis between PD and AD risk. In MR between α-synuclein in PD with AD risk, we only extracted rs356182 as the IV through a strict screening process. The result indicated a significant association based on IVW method (OR = 0.638, 95% CI: 0.485–0.838, *P* = 1.20E-03). In order to examine the robustness of the IVW method, we used other three complementary analytical methods and also obtained consistent results.

**Conclusion:**

The overall PD genetic risk factors did not predict AD risk, but the α-synuclein susceptibility genetic variants in PD reduce the AD risk. We believe that our findings may help to understand the association between them, which may be useful for future genetic studies for both diseases.

**Electronic supplementary material:**

The online version of this article (10.1186/s12881-018-0721-7) contains supplementary material, which is available to authorized users.

## Background

Alzheimer’s disease (AD) and Parkinson’s disease (PD) are the top two common neurodegenerative diseases [1]. In 2015, AD and PD respectively affected about 29.8 million and 6.2 million people worldwide [2]. AD is characterized by a progressive decline in memory, language, problem solving and other cognitive functions [3]. The neuropathological changes of AD are mainly manifested as brain atrophy and the accumulation of abundant extracellular Aβ plaques and intraneuronal neurofibrillary tau tangles [4, 5]. The patients suffering from PD show slow movements, tremor, gait and balance disturbances and other behavioral problems [6]. The main pathological characteristics of PD are the progressive degeneration of dopaminergic neurons in the substantia nigra and the presence of Lewy bodies, which are made of abnormal filaments composed of α-synuclein [7, 8].

The two neurodegenerative diseases share many clinical and pathological features. Because of the toxicity of the α-synuclein or Aβ42 and tau proteins, so many similar cascades of neuronal reactions leading to progressive neurodegeneration occur in PD and AD patients. The overlapping pathological changes of AD and PD include activation of glycogen synthase kinase-3 beta, mitogen-activated protein kinases, cell cycle reentry and oxidative stress, etc. [9]. As we all know, mild cognitive impairment (MCI) is associated with the risk of progression to AD. However, MCI was recently also recognized as a kind of subtype predicting progression to PD [10, 11]. About 25% of non-demented patients with PD meet neuropsychological test criteria for MCI [12] and more than 50% of them eventually develop dementia [11]. All of these suggest that the two neurodegenerative diseases are probably the risk factor of each other.

In addition to the similarities between AD and PD, some potential mechanisms linking these two neurodegenerative diseases were found recently, especially the α-synuclein and Lewy pathology in AD. Several studies found that up to 50% AD patients exhibited extra aggregation of α-synuclein into Lewy bodies [13, 14]. Statistic test did not identify any significant difference of α-synuclein levels in cerebrospinal fluid between AD and PD patients [15]. In transgenic mice, the accumulation of α-synuclein could significantly disrupt cognition [14]. A similar study found that the α-synuclein in the transgenic mice could also massive increase apolipoprotein E (ApoE) levels and accelerate the accumulation of insoluble mouse Aβ [16]. Furthermore, the cerebrospinal fluid total α-synuclein levels contributed to the differential diagnosis of AD versus other dementias [17]. In the familial AD patients with presenilin 1 (PSEN1) mutations, which is most commonly associated with familial forms of AD, the notable interaction between α-synuclein and PSEN1 was identified in post-mortem brain tissue [9]. Our previously study conducted an interaction network using genome-wide association studies (GWASs) data and found the significant interaction among AD and PD susceptibility genes [7]. All of these evidences suggest a further investigation for the potential genetic mechanisms linking PD to AD, particularly the role that the α-synuclein plays in AD.

In recent years, large-scale GWAS identified some common genetic variants and provided insights into the genetics of AD and PD, including α-synuclein coding gene Synuclein Alpha (SNCA) ^18,19^. The large-scale GWAS datasets provide tremendous support for investigating the potential genetic association between PD and AD risk by Mendelian randomization analytical method (MR). MR is conceptually similar to randomized controlled trials (RCT) and can be applied to investigate the causality of biomarkers in disease etiology based on GWAS summary data^20,21^. In our study, we conducted a MR analysis with the lead single nucleotide polymorphisms (lead SNPs, which showed the strongest association in GWAS) from PD susceptibility loci. Here, these lead SNPs defined as instrumental variable (IV). In the results of sensitivity analysis of the MR between PD and AD risk, we found that the absence of SNCA lead SNP would massive decrease the statistical significance. Considering the reported role of α-synuclein in AD pathology, we further performed a MR to investigate the genetic association of the α-synuclein and AD risk.

## Materials and methods

### Study design

**Fig. 1:**
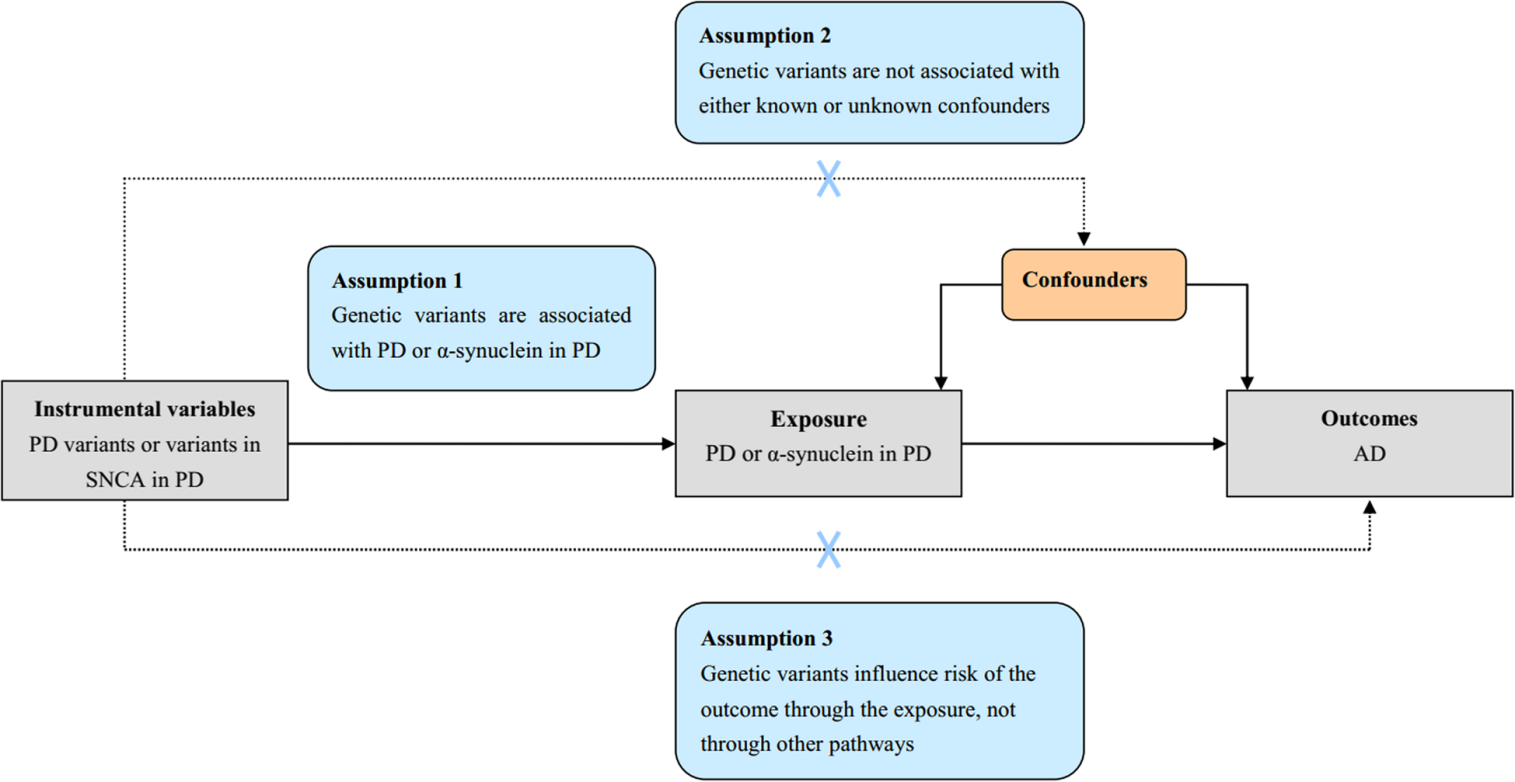
Mendelian randomization assumptions. The Mendelian randomization is based on three principal assumptions, which have been widely described in recent studies [18, 29]. First, the genetic variants selected to be IVs should be associated with the exposure (PD or α-synuclein in PD) (assumption 1) [18, 29]. Second, the genetic variants should not be associated with confounders (assumption 2) [18, 29]. Third, genetic variants should affect the risk of the outcome (AD) only through the exposure (PD or α-synuclein in PD) (assumption 3) [18, 29]

MR is based on the premise that the human genetic variants are randomly distributed in the population [18, 19]. These genetic variants are largely not associated with confounders and can be used as IVs to estimate the causal association of exposures (PD, α-synuclein in PD) with an outcome (AD) [18, 19]. The MR is based on three principal assumptions (Fig. 1), which have been widely described in recent studies [18, 19]. First, the genetic variants selected to be IVs should be associated with the exposures (PD, α-synuclein in PD) (assumption 1 in Fig. 1) ^20,22^. Second, the genetic variants should not be associated with confounders (assumption 2 in Fig. 1) [18, 19]. Third, genetic variants should affect the risk of the outcome (AD) only through the exposure (PD or α-synuclein in PD) (assumption 3 in Fig. 1) ^20,22^. The second and third assumptions are collectively known as independence from pleiotropy [18, 19]. This study is based on the publicly available, large-scale GWAS summary datasets, and the genetic variants are SNPs. All participants gave informed consent in all these corresponding original studies.

### PD GWAS dataset

Here, we first used PD susceptibility SNPs as the IVs that from the most large-scale PD GWAS dataset [20]. This GWAS included 428,235 individuals (26,035 PD cases and 403,190 controls) consisting of 416,518 individuals in discovery stage and 21,679 individuals in replication stage [20]. The final joint analysis identified 17 novel loci associated with PD with the genome-wide significance (*P* < 5.00E-08) [20]. We extracted lead SNPs information of each locus from the joint analysis results except rs601999, which did not provide sufficient data. We also obtained 27 genome-wide significant loci from other more earlier GWASs^23,24^. But the basic lead SNPs information of these 27 loci were extracted from the discovery stage of the most large-scale GWAS [19]. All these 43 lead SNPs were independent [20].

### Reported SNCA mutations in PD

We acquired the PD susceptibility SNCA lead SNPs through a rigorous screening process. We first searched all previous GWASs which reported SNCA as a PD susceptibility locus. Afterwards, we obtained the lead SNPs from each GWAS. However, we would exclude the lead SNP when they were not reported by GWAS of European ethnicity. When a lead SNP is reported by more than one GWAS, we would extract the summary statistics from the most large-scale GWAS because of its greater statistic power. When several lead SNPs were in linkage disequilibrium (LD), we would choose the one from the most large-scale GWAS. We searched the LD information by using the HaploReg v4.1, which based on LD data from 1000 Genomes Project (CEU) [21].

Finally, we only selected one lead SNP from 7 unduplicated lead SNPs. The sources and the detailed information about the 7 SNPs are described in Table 1. We excluded the SNPs rs11931074, rs8180209 and rs6532194 because they were reported in GWASs of Asian ethnicity. We then found that all of the rest 4 SNPs were located in SNCA. Considering the rs356182 was overwhelmingly significantly associated with PD risk in the most large-scale PD GWAS [20], we selected this genetic variant as tagged SNP to perform MR analysis.

**Table 1:** Characteristics of 7 SNCA genetic variants reported in PD GWASs

SNP	Chr:BP^a^	Reported year	Source	Reported ancestry	LD (r^2^)^b^	Function class	EA	NEA	EAF	OR (95% CI)	*P* value
rs356182	4: 90626111	2017	Chang D [20]	European	1	Intron variant	G	A	0.404	1.33 (1.30–1.36)	5.21E-123
rs356219	4:89716450	2012	Lill CM [47]	Caucasian	0.76	Intron variant	G	A	0.41	1.29 (1.25–1.33)	6.00E-65
rs356220	4:89720189	2014	Hill-Burns EM [48]	European	0.51	Intron variant	T	C	0.364	1.38 (1.24–1.52)	3.00E-11
rs2736990	4:89757390	2010	Edwards DL [49]	Caucasian	0.48	Intron variant	G	A	0.52	1.30 (1.18–1.43)	6.74E-8
rs8180209	4:89723303	2017	Foo JN [50]	Han Chinese	NA^c^	Intron variant	A	G	0.07	0.41 (NA^d^)	1.02E-32
rs11931074	4:89718364	2009	Satake W [51]	Japanese	NA^c^	Intron variant	G	T	0.36	1.37 (1.27–1.48)	7.35E-17
rs6532194	4:89859751	2012	Lill CM [47]	Asian	NA^c^	Intergenic variant	T	C	0.40	1.29 (1.20–1.39)	4.91E-11

^a^Chr:BP, Chromosome:Position

^b^represent the r^2^ value of linkage disequilibrium (LD) between the selected genetic variant and the tagged mutation rs356182. The range of r^2^ is 0–1, the greater the value of r^2^, the stronger the linkage disequilibrium

^c^The genetic variants were not reported in European (or Caucasian) populations

^d^The 95% CI of OR was not available

### AD GWAS dataset

The AD GWAS dataset was downloaded from the large-scale meta-analysis, which was performed by the International Genomics of Alzheimer’s Project (IGAP) [22]. In stage 1, the IGAP genotyped and imputed 7,055,881 SNPs, and performed a meta-analysis of four GWAS datasets including 17,008 cases and 37,154 controls of European descent [22]. All patients with AD satisfied the NINCDS-ADRDA criteria or DSM-IV guidelines [22]. Here, we would extract the summary statistics of the 43 lead SNPs from stage 1 results of the AD GWAS.

### Pleiotropy analysis

In MR study, there is a potential violation of assumption 2 and 3 through pleiotropy that a genetic instrument is associated with a study outcome through biological pathways outside the interest exposure. Here, we performed an assessment for pleiotropy to assure that all the selected genetic variants do not exert effects on AD risk through biological pathways independent of PD pathological changes. Five steps were taken to reduce the risk of pleiotropy. In stage 1, nine potentially modifiable risk factors, which were reported in a major review about dementia risk factors, were considered [23]. The nine risk factors consisted of low levels of education, midlife hearing loss, physical inactivity, high blood pressure (hypertension), type 2 diabetes, obesity, smoking, depression, and social isolation [23]. We excluded obesity because a recent MR study reported that this trait as well as body mass index (BMI) is not genetically associated with AD risk [24]. In stage 2, we investigated whether the dietary factors are the potential confounders [23, 25]. The UK Biobank datasets were available [26]. Some studies reported that alcohol play an important role in AD risk, so we considered it as a potential confounder in stage 3 [27]. In stage 4, we evaluated the potential pleiotropic associations of IVs with AD biomarkers, which contain cerebrospinal fluid tau, phosphorylated tau (ptau) and Aβ42 [28]. In last stage, we selected MR-Egger intercept test to evaluate the potential pleiotropic associations of these genetic variants with potential confounders. Here, we provided more detailed information about the selected exposures and counterpart GWASs are described in Additional file 1.

### Mendelian randomization analysis

We conducted two MR analyses to investigate the genetic association between PD and AD risk and the genetic association between α-synuclein in PD and AD risk. Until now, four different MR analytical methods have been well established including inverse-variance weighted meta-analysis (IVW), weighted median regression, MR-Egger regression and maximum-likelihood regression [18, 29]. However, the IVW has more statistical power than the other three methods [30]. The detailed information of IVW were described in Additional file 2. Particularly, MR-Egger regression always including a wide confidence intervals even the null hypothesis [30]. Therefore, we selected the IVW as the main analytical method to perform the MR analysis, as did in previous MR studies [19, 29].

Besides, in MR analysis of PD and AD risk, we also selected the weighted median regression, MR-Egger regression and maximum-likelihood method as the complementary analytical methods to examine the robustness of the IVW estimate. MR-Egger could also provide a statistical test the presence of potential pleiotropy, and account for this potential pleiotropy [31]. If there was no clear evidence of pleiotropy, we would expect all the four tests to give consistent estimates. We also performed a sensitivity analysis using the leave-one-out permutation method. In the MR analysis between α-synuclein in PD and AD risk, we only selected maximum-likelihood method as complementary analytical method because of the quantitative limitation of IV.

All analyses were conducted using the R package ‘MendelianRandomization’ [32]. We choose *P* < 0.05 as discriminant criterion for statistical significant result of the MR study.

## Results

### Instrumental variable

In MR analysis between PD and AD risk, we obtained 43 SNPs associated with PD as potential IVs. However, one of these 43 genetic variants as well as its proxy genetic variants were not available in stage 1 dataset of AD GWAS. The genetic variant was rs143918452 and we excluded it from the IVs. All the rest SNPs could map to genes implicated in PD pathology pathways, including autophagy, mitochondria, immune system, lysosome and endocytosis, etc. [20, 33, 34].

In MR analysis between α-synuclein in PD and AD risk, we only selected rs356182 after a rigorous screening process. As we all know, α-synuclein plays an important role in almost all PD pathology pathways. The genetic variants at SNCA locus (coding α-synuclein) can alter the risk of PD and even lead to monogenic and severe early-onset forms PD [34]. The rs356182 were reported as lead SNP of SNCA locus in most PD GWASs, which was also the strongest association signal in the most large-scale PD GWAS [20]. Recently, some studies found that rs356182 was associated with a specific SNCA 5′ untranslated region transcript isoform and regular the SNCA expression in brain [35, 36]. The firm and reliable association between rs356182 and α-synuclein make the SNP perfectly meet the requirements of IV.

### Pleiotropy of instrumental variables

We identified that rs11343 and rs17649553 were significantly associated with years of education in stage1 (*P* = 1.20E-4 and *P* = 2.33E-07 respectively). We also found that rs17649553 was associated with Vitamin in stage 2 and continuous alcohol in stage 3. In stage 2, rs9275326 was associated with vitamin C, vitamin B9 and mineral supplements with *P* = 6.42E-04, 1.95E-09 and 2.63E-06. There were no genetic variants associated with AD biomarker. In stage 5, the MR-Egger intercept test showed no significant intercept (MR-Egger intercept β = 0.003; *P* = 0.717) in these genetic variants. By iteratively pruning the corresponding variant list, we also didn’t find any significant intercept. The detailed information about the pleiotropy of these genetic variants is described in Table S1-S3 in Additional file 3 and Table S4 in Additional file 4.

In summary, our pleiotropy analysis showed that rs11343, rs17649553 and rs9275326 may have potential violation of assumption 2 and 3 through pleiotropy. To meet the MR assumptions (Fig. 1), we excluded these variants in following analyses. The rest 39 genetic variants which used in following analyses were detailed described in Table 2.

**Table 2:** Characteristics of 39 genetic variants in PD and AD GWAS datasets

SNP	Chr	Pos	Nearby Genes	EA	NEA	EAF^a^	PD			AD GWAS		
							Beta^b^	SE	*P* value	Beta^b^	SE	*P* value
rs10797576	1	232,664,611	SIPA1L2	T	C	0.127	0.113	0.014	8.41E-13	0.006	0.022	0.795
rs10906923	10	15,569,598	FAM171A1	A	C	0.461	0.073	0.014	1.35E-8	−0.033	0.017	0.051
rs11060180	12	123,303,586	OGFOD2	A	G	0.748	0.105	0.011	2.05E-20	0.032	0.019	0.081
rs11158026	14	55,348,869	GCH1	C	T	0.490	0.094	0.011	4.30E-16	−0.002	0.017	0.894
rs115185635	3	87,520,857	CHMP2B	C	G	0.012	0.191	0.048	1.22E-4	−0.022	0.058	0.702
rs11724635	4	15,737,101	FAM200B, CD38	A	C	0.408	0.105	0.011	1.22E-19	−0.032	0.016	0.040
rs117896735	10	121,536,327	BAG3	A	G	0.004	0.501	0.057	2.23E-19	0.133	0.086	0.119
rs12456492	18	40,673,380	SYT4	G	A	0.330	0.095	0.012	5.56E-16	0.009	0.017	0.589
rs12497850	3	48,748,989	NCKIPSD, CDC71	T	G	0.731	0.073	0.014	9.16E-9	0.028	0.017	0.088
rs12637471	3	182,762,437	MCCC1	G	A	0.663	0.163	0.015	2.11E-30	0.002	0.019	0.909
rs13294100	9	17,579,690	SH3GL2	G	T	0.457	0.083	0.014	4.84E-13	0.013	0.017	0.453
rs14235	16	31,121,793	ZNF646, KAT8	A	G	0.359	0.077	0.009	5.44E-12	0.041	0.016	0.011
rs1474055	2	169,110,394	STK39	T	C	0.201	0.186	0.018	5.68E-26	0.004	0.024	0.881
rs1555399	14	67,984,370	TMEM229B	T	A	0.607	0.086	0.012	9.61E-11	−0.021	0.016	0.194
rs199347	7	23,293,746	KLHL7, NUPL2, GPNMB	A	G	0.483	0.094	0.011	3.51E-18	−0.033	0.016	0.039
rs2280104	8	22,525,980	SORBS3, PDLIM2, C8orf58, BIN3	T	C	0.265	0.068	0.012	2.53E-8	−0.002	0.016	0.923
rs2414739	15	61,994,134	VPS13C	A	G	0.679	0.094	0.011	3.94E-14	−8.00E-04	0.017	0.964
rs2694528	5	60,273,923	ELOVL7	C	A	0.135	0.140	0.020	4.84E-15	−0.028	0.027	0.305
rs2740594	8	11,707,174	CTSB	A	G	0.893	0.086	0.012	5.91E-12	0.002	0.018	0.926
rs329648	11	133,765,367	MIR4697	T	C	0.465	0.086	0.012	1.11E-13	−0.007	0.018	0.701
rs34043159	2	102,413,116	IL1R2	C	T	0.332	0.077	0.009	5.48E-11	0.006	0.016	0.695
rs34311866	4	951,947	TMEM175,DGKQ	C	T	0.140	0.207	0.014	1.47E-50	0.009	0.021	0.685
rs353116	2	166,133,632	SCN3A	C	T	0.557	0.062	0.011	2.98E-8	0.038	0.016	0.021
rs356182	4	90,626,111	SNCA	G	A	0.404	0.285	0.012	5.21E-123	−0.056	0.017	0.001
rs35749011	1	155,135,036	GBA	A	G	0.005	0.545	0.044	2.59E-35	0.188	0.071	0.008
rs3793947	11	83,544,472	DLG2	G	A	0.570	0.073	0.011	3.72E-9	−0.010	0.016	0.525
rs4073221	3	18,277,488	SATB1	G	T	0.062	0.095	0.016	1.57E-8	0.007	0.023	0.754
rs4653767	1	226,916,078	ITPKB	T	C	0.726	0.083	0.011	1.63E-11	−0.005	0.017	0.756
rs4784227	16	52,599,188	TOX3	T	C	0.200	0.086	0.014	9.75E-11	−0.010	0.018	0.580
rs591323	8	16,697,091	MICU3	G	A	0.635	0.094	0.014	2.38E-11	−0.002	0.018	0.930
rs62120679	19	2,363,319	LSM7	T	C	0.426	0.077	0.014	6.64E-7	−0.004	0.022	0.843
rs6430538	2	135,539,967	TMEM163,CCNT2	C	T	0.189	0.117	0.011	8.24E-24	−0.047	0.017	0.006
rs6812193	4	77,198,986	FAM47E	C	T	0.687	0.083	0.011	1.43E-14	−0.017	0.016	0.310
rs76904798	12	40,614,434	LRRK2	T	C	0.132	0.140	0.015	1.21E-19	−0.016	0.022	0.483
rs78738012	4	114,360,372	ANK2, CAMK2D	C	T	0.039	0.122	0.018	4.78E-11	−0.012	0.029	0.680
rs8005172	14	88,472,612	GALC	T	C	0.434	0.077	0.012	8.77E-11	−0.021	0.016	0.171
rs8118008	20	3,168,166	DDRGK1	A	G	0.540	0.068	0.012	1.99E-6	0.014	0.018	0.432
rs823118	1	205,723,572	NUCKS1, SLC41A1	T	C	0.411	0.117	0.011	1.12E-23	−0.003	0.016	0.846
rs9468199	6	27,681,215	ZNF184	A	G	0.300	0.104	0.014	1.46E-12	0.044	0.022	0.046

*SNP* single-nucleotide polymorphism, *Chr* Chromosome, *Pos* Position, *EA* Effect Allele, *NEA* Non-Effect Allele, *EAF* Effect Allele Frequency, *PD* Parkinson’s disease, *AD* Alzheimer’s disease, *GWAS* genome-wide association studies, *SE* standard error

^a^Frequency of the effect allele in 1000 Genomes Project (CEU)

^b^Beta is the regression coefficient based on the effect allele. Beta > 0 and Beta < 0 means that this effect allele regulates increased and reduced PD or AD risk, respectively

### Mendelian randomization between PD and AD risk

**Fig. 2:**
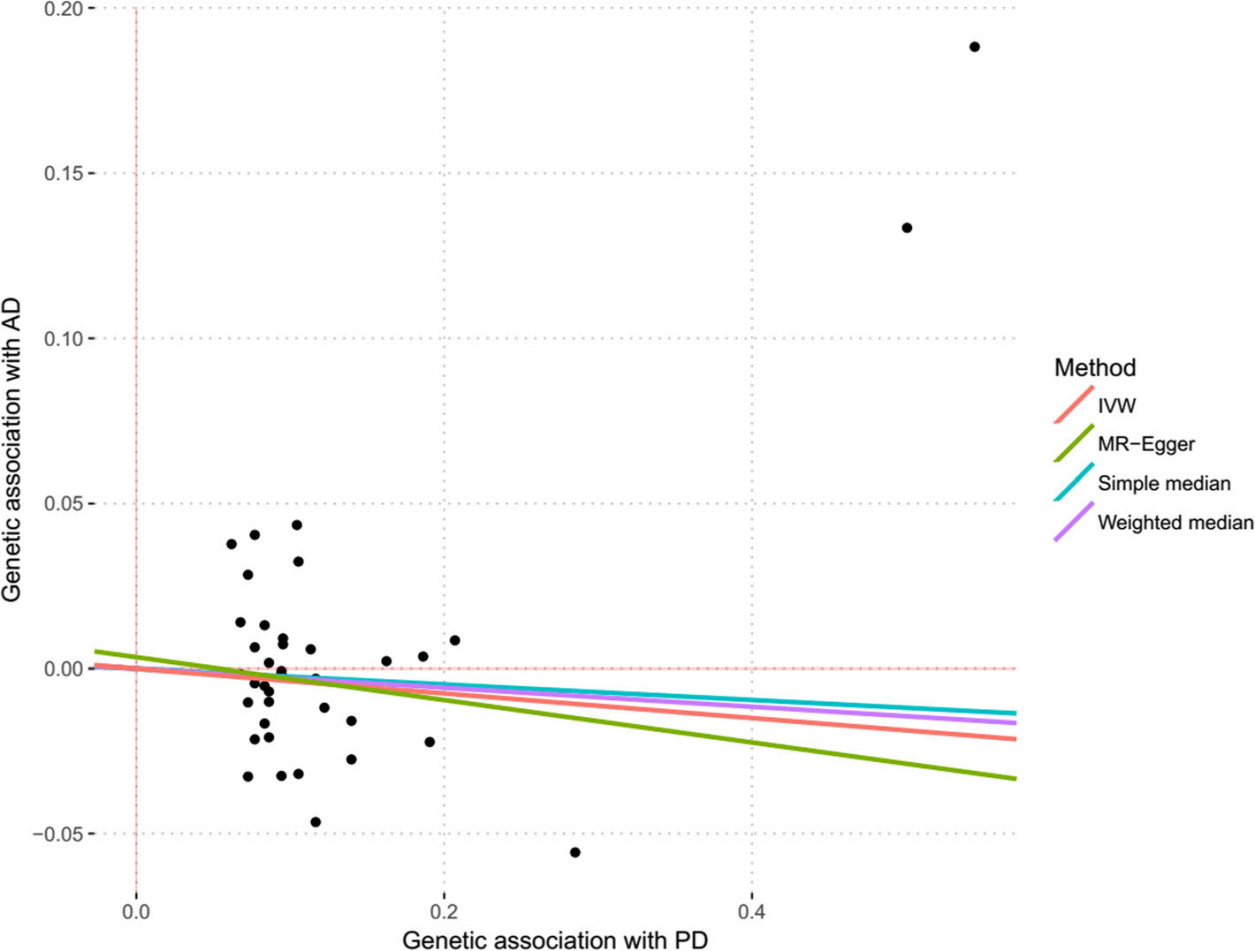
Individual genetic estimates from each of 39 genetic variants using different methods. This scatter plot show individual causal estimates from each of 6 genetic variants associated with PD on the x-axis and AD risk on the y-axis. The continuous line represents the causal estimate of PD on AD risk

Using the remaining 39 genetic variants, IVW analysis showed that the genetically predicted PD was not significantly associated with AD risk (OR = 0.918, 95% CI: 0.782–1.076, *P* = 0.291). Furthermore, all of the weighted median estimate (OR = 1.069, 95% CI: 0.769–1.140, *P* = 0.509), MR-Egger estimate (OR = 0.863, 95% CI: 0.593–1.253, *P* = 0.437) and maximum-likelihood estimate (OR = 0.916, 95% CI: 0.780–1.076, *P* = 0.292) were consistent with the IVW estimate in terms of direction and magnitude. Meanwhile, the MR-Egger intercept test showed no significant intercept (β = 0.003, *P* = 0.717) between these 39 variants. Figure 2 shows individual genetic estimates from each of the 39 genetic variants using different methods.

### Sensitivity analysis between PD and AD risk

To further test the stability of these estimates, we sequentially removed each genetic variant in the MR analysis. The direction and precision of the genetic estimates between PD and the risk of AD remained largely unchanged using these MR methods except maximum-likelihood method (Table 3). In spite the exclusion of rs356182 notably increased the significance of the association between PD and AD risk, it is *P* value still not reach 0.05.

**Table 3:** sensitivity analysis of the association between PD and AD risk

SNP^a^	*P* value			SNP^a^	*P* value		
	IVW^b^	Wei-Med^c^	MR-Egger^d^		IVW^b^	Wei-Med^c^	MR-Egger^d^
rs10797576	0.281	0.600	0.437	rs34043159	0.277	0.596	0.465
rs10906923	0.365	0.653	0.348	rs34311866	0.245	0.608	0.346
rs11060180	0.200	0.598	0.422	rs353116	0.204	0.593	0.606
rs11158026	0.299	0.660	0.444	rs356182	0.991	0.649	0.313
rs115185635	0.307	0.654	0.454	rs35749011	0.117	0.592	0.075
rs11724635	0.419	0.653	0.441	rs3793947	0.320	0.651	0.418
rs117896735	0.207	0.597	0.240	rs4073221	0.283	0.594	0.446
rs12456492	0.264	0.599	0.450	rs4653767	0.307	0.653	0.438
rs12497850	0.222	0.594	0.527	rs4784227	0.318	0.652	0.433
rs12637471	0.277	0.608	0.411	rs591323	0.297	0.596	0.444
rs13294100	0.260	0.597	0.468	rs62120679	0.302	0.649	0.440
rs14235	0.172	0.593	0.547	rs6430538	0.473	0.648	0.482
rs1474055	0.277	0.605	0.406	rs6812193	0.340	0.653	0.414
rs1555399	0.355	0.652	0.408	rs76904798	0.331	0.656	0.465
rs199347	0.404	0.651	0.405	rs78738012	0.310	0.652	0.448
rs2280104	0.299	0.647	0.445	rs8005172	0.353	0.652	0.385
rs2414739	0.295	0.599	0.445	rs8118008	0.267	0.594	0.490
rs2694528	0.341	0.654	0.474	rs823118	0.301	0.662	0.442
rs2740594	0.290	0.597	0.449	rs9468199	0.201	0.596	0.422
rs329648	0.311	0.654	0.437				

^a^the SNP that was excluded in sensitivity analysis

^b^IVW, Inverse-variance weighted meta-analysis

^c^Wei-Med, Weighted median

^d^MR-Egger, MR-Egger regression

### Mendelian randomization between α-synuclein in PD and AD risk

Using rs356182 as IV, IVW analysis showed that the genetically changed PD α-synuclein was significantly associated with a reduced AD risk (OR = 0.638, 95% CI: 0.485–0.838, *P* = 1.20E-03). The maximum-likelihood estimate (OR = 0.60, 95% CI: 0.34–1.06, *P* = 0.08) was completely consistent with the IVW estimate.

## Discussion

In this study, we firstly selected 39 SNPs as IVs to investigate the association between PD and AD risk by using MR methods. However, we didn’t identify any significant results with all *P* values greater than 0.2. In the sensitivity analysis, we found that in the absence of rs356182, the *P* value of the IVW result rose to nearly 1. Considering some PD patients exhibited AD characteristics as described in the introduction, we secondly selected rs356182 as IV and conducted another one MR. The results of this MR indicated that the α-synuclein in PD was significantly associated with AD risk. The consistent results of different MR methods increased the credibility of our findings.

Besides the similar clinical and neuropathological similarities as we all know, amounts of genetic overlap between AD and PD were also discovered^42,43^. The evaluation of the α-synuclein functional characterization in AD can be traced back to the last century [37]. From then on, a lot of evidences supported the discovery as described in introduction. Recently, many studies have found the potential genetic association between AD and PD [38–42]. A trans-pQTL study discovered the PD risk variant rs12456492 affects expression of AD-associated protein CD33 in peripheral monocytes [41]. Genetic association study proved rs76904798 of LRRK2 significantly reduce late-onset AD risk in Han Chinese [43]. The AD risk factor SORL1 had been identified to be associated with PD recently [38].

The rs356182 locates in the intron of SNCA, which is the coding gene of α-synuclein. In addition to the key role of α-synuclein in PD, rs356182 can defines PD endophenotype in different levels [36, 44, 45]. In our study, the association between α-synuclein and AD identified by rs356182 as IV greatly supported and extended the genetic overlap of AD and PD. The effect size of the IV was the regression coefficient between SNCA loci (the counts of effect allele) and PD. Therefore our results suggested that the α-synuclein function in PD probably causes AD pathological changes. This conclusion supported the phenomenon that some PD patients meet neuropsychological test criteria for MCI [12].

But the overall genetic risk factors of PD were not statistically associated with AD risk in our study. It can’t be ignored that PD is a complex disease with so many pathogenic factors leading to kinds of pathological changes^19,41^. However, there may be only one kind of the PD pathological changes is genetically associated with AD risk. And including all the PD pathogenic factors will reduce or even eliminate this genetic association statistically. A similar MR study investigated the association between type 2 diabetes (T2D) and AD [46]. They chose mechanism-specific genetic variants as IVs to overcome this problem [46]. And they found only insulin sensitivity genetic variants in T2D are associated with AD risk (OR = 1.17; 95% CI: 1.02–1.34) [46]. However, there are ambiguous boundaries between different pathogenic mechanisms in PD. Furthermore, one genetic variant is probably involved in many pathological pathways. That means the IVs of the mechanism-specific in PD may violate the MR assumptions. So we adopted the molecular-specific genetic variants as the IV in our study.

Nevertheless, we still could not exclude all the other possible pathways besides the PD pathological pathway that α-synuclein play roles in AD. In other words, the potential pleiotropy of the rs356182 may be the biggest problem in the study. That’s the insufficient of MR and selecting one genetic variant as IV expands this deficiency. Another limitation of our study is the absence of a well PD pathogenic mechanism division method. Overcoming these problems will greatly improve our understanding about the effect of PD pathological changes on AD risk.

## Conclusions

As we all know, PD and AD have many clinical and pathological features. However, there is little genetic proof for the association between these neurodegenerative diseases. In this MR study, we investigated the genetic roles of PD and its molecular pathological changes in AD. The results indicated only the α-synuclein in PD was genetically associated with AD risk. The pleiotropy analysis demonstrated all of the 39 PD susceptibility SNPs used as IVs were not linked with any reported AD risk factors. Future studies could identify the potential biological process that linking the α-synuclein and AD. We believe this finding will help to understand the pathology of AD and PD, which will stimulate subsequent research.

## Additional files

Additional file 1: A more detailed description about the selected exposures and counterpart GWASs. And how we conducted the pleiotropy analysis with these datasets. (DOCX 95 kb)

Additional file 2: Statistical description of Inverse-variance weighted method. (DOCX 71 kb)

Additional file 3: The genetic association between PD susceptibility SNPs with potential AD risk factors. (XLSX 18 kb)

Additional file 4: The results of the MR-Egger intercept test. (XLSX 10 kb)

## Abbreviations

AD: Alzheimer’s disease
CI: Confidence interval
GWAS: Genome-wide association study
IGAP: International Genomics of Alzheimer’s Project
IV: Instrumental variable
IVW: Inverse-variance weighted meta-analysis
LD: Linkage disequilibrium
MCI: Mild cognitive impairment
MR: Mendelian randomization
OR: Odd ratio
PD: Parkinson’s disease
pQTL: Protein quantitative trait loci
RCT: Randomized controlled trial
SNP: Single nucleotide polymorphism
T2D: Type 2 diabetes
