# Supplementary material for: Parkinson’s disease and Alzheimer’s disease: a Mendelian randomization study
Source: BMC Med Genet. 2018 Dec 31;19(Suppl 1):215. doi: 10.1186/s12881-018-0721-7 (PMC6311900; doi:10.1186/s12881-018-0721-7)
Supplement: Supplementary file 1 — A more detailed description about the selected exposures and counterpart GWASs. And how we conducted the pleiotropy analysis with these datasets. (DOCX 95 kb) [file 12881_2018_721_MOESM1_ESM.docx]

**Pleiotropy Analysis**

In Mendelian randomization study, one important issue is the potential violation of assumption 2 and 3 through pleiotropy occurring when a genetic instrument is associated with a study outcome through biological pathways outside the exposure of interest. Here, we performed an assessment for pleiotropy to assure that the selected genetic variants do not exert effects on AD risk through biological pathways independent of serum calcium levels. A number of steps were taken to reduce the risk of pleiotropy.

In 2017, a major review by The Lancet has identified nine potentially modifiable risk factors linked to dementia^1^. This review suggests that around 35% of dementia is attributable to a combination of these nine risk factors^1^. In stage 1, we referred the major review and evaluated the potential pleiotropy using eight known confounders including years of educational attainment from Social Science Genetic Association Consortium (SSGAC)^2^, type 2 diabetes from DIAbetes Genetics Replication and Meta-analysis (DIAGRAM) Consortium^3^, cigarettes smoked per day from the Tobacco and Genetics Consortium (TGC) ^4^, major depressive disorder from Psychiatric Genomics Consortium (PGC)^5^, blood pressure including systolic blood pressure (SBP) and diastolic blood pressure (DBP) from the International Consortium of Blood Pressure (ICBP)^6^, hearing loss from UK biobank^7^.

In stage 2, we evaluated the potential pleiotropic associations of these 43 variants with mineral supplements (including calcium, fish oil (including cod liver oil), glucosamine, iron, selenium, zinc and others), and vitamin supplements (including folic acid or folate (Vit B9), vitamin A, vitamin B, vitamin C, vitamin D, vitamin E, multivitamins and others)^7^. We then particularly investigated continuous alcohol and dichotomous alcohol as potential confounder^8^.

In stage 4, we obtained the summary data of Aβ42, tau, and ptau levels in cerebrospinal fluid (CSF) from an Alzheimer's endophenotypes and disease modifiers GWA study containing 3146 particpants^9^. In stage 1-4, the significance threshold for the association of these 43 variants with these known and potential confounders is P < 1.16E-3 (a Bonferroni correction, P < 0.05/43).

In addition to the known confounders above, there may also be some unknown confounders. In stage 5, we selected a statistical method to evaluate the potential pleiotropic associations of these 39 genetic variants with known and unknown confounders. The method is MR-Egger intercept test, which could provide an assessment of the validity of the instrumental variable assumptions, and provide a statistical test the presence of potential pleiotropy^10^. Here, we used the method to iteratively prune the corresponding variant list until there is pleiotropy, which allows for an agnostic assessment of pleiotropy caused by both known and unknown confounders^11^.

**Reference**

1 Livingston, G. *et al.* Dementia prevention, intervention, and care. *Lancet* **390**, 2673-2734, doi:10.1016/S0140-6736(17)31363-6 (2017).

2 Okbay, A. *et al.* Genome-wide association study identifies 74 loci associated with educational attainment. *Nature* **533**, 539-542, doi:10.1038/nature17671 (2016).

3 Replication, D. I. G. *et al.* Genome-wide trans-ancestry meta-analysis provides insight into the genetic architecture of type 2 diabetes susceptibility. *Nature genetics* **46**, 234-244, doi:10.1038/ng.2897 (2014).

4 Tobacco & Genetics, C. Genome-wide meta-analyses identify multiple loci associated with smoking behavior. *Nature genetics* **42**, 441-447, doi:10.1038/ng.571 (2010).

5 Major Depressive Disorder Working Group of the Psychiatric, G. C. *et al.* A mega-analysis of genome-wide association studies for major depressive disorder. *Molecular psychiatry* **18**, 497-511, doi:10.1038/mp.2012.21 (2013).

6 International Consortium for Blood Pressure Genome-Wide Association, S. *et al.* Genetic variants in novel pathways influence blood pressure and cardiovascular disease risk. *Nature* **478**, 103-109, doi:10.1038/nature10405 (2011).

7 Sudlow, C. *et al.* UK biobank: an open access resource for identifying the causes of a wide range of complex diseases of middle and old age. *PLoS medicine* **12**, e1001779, doi:10.1371/journal.pmed.1001779 (2015).

8 Schumann, G. *et al.* KLB is associated with alcohol drinking, and its gene product beta-Klotho is necessary for FGF21 regulation of alcohol preference. *Proceedings of the National Academy of Sciences of the United States of America* **113**, 14372-14377, doi:10.1073/pnas.1611243113 (2016).

9 Deming, Y. *et al.* Genome-wide association study identifies four novel loci associated with Alzheimer's endophenotypes and disease modifiers. *Acta neuropathologica* **133**, 839-856, doi:10.1007/s00401-017-1685-y (2017).

10 Tillmann, T. *et al.* Education and coronary heart disease: mendelian randomisation study. *Bmj* **358**, j3542, doi:10.1136/bmj.j3542 (2017).

11 Burgess, S. & Thompson, S. G. Interpreting findings from Mendelian randomization using the MR-Egger method. *European journal of epidemiology* **32**, 377-389, doi:10.1007/s10654-017-0255-x (2017).
