# Supplementary material for: Parkinson’s disease and Alzheimer’s disease: a Mendelian randomization study
Source: BMC Med Genet. 2018 Dec 31;19(Suppl 1):215. doi: 10.1186/s12881-018-0721-7 (PMC6311900; doi:10.1186/s12881-018-0721-7)
Supplement: Supplementary file 2 — Statistical description of Inverse-variance weighted method. (DOCX 71 kb) [file 12881_2018_721_MOESM2_ESM.docx]

**Assumed framework of data and genetic Associations**

Suppose that we have selected J independent genetic variants $G_{j}$($j=1,...,J$) to act as instruments in a two-sample Mendelian randomization study. Here, we have successfully extracted the summary results about the associations of each genetic variant $G_{j}$($j=1,...,J$) with the risk factor (or with each risk factor for the multivariable setting) and with the outcome including the beta coefficients ($\beta^{\wedge} _{Xj}$,$\beta^{\wedge} _{Yj}$) and their standard errors ($se(\beta^{\wedge} _{Xj})$, and$se(\beta^{\wedge} _{Yj})$). We assume throughout that the parametric assumptions of linearity with no effect heterogeneity hold for the causal exposure-outcome relationship, and for the instrumental variable-exposure and instrumental variable-outcome associations for all instruments.

Initially, we consider the causal effect of a risk factor X on an outcome Y using genetic variants $G_{j}$($j=1,...,J$) that are assumed to be uncorrelated (not in linkage disequilibrium) [1]. The association between genetic variant $G_{j}$($j=1,...,J$) and the outcome is denoted$\beta_{Yj}$, and the association between genetic variant $G_{j}$ and the risk factor is denoted$\beta_{Xj}$[1]. The genetic association with the outcome can be decomposed into the sum of a direct (pleiotropic) effect and an indirect (causal) effect:

$$\beta_{Y_{j}}=\alpha_{j}+\theta\beta_{Xj}$$

where $\alpha_{j}$is the effect of the genetic variant on the outcome that is not mediated via the risk factor of interest, and $\theta$ is the causal effect of the risk factor on the outcome. A genetic variant is referred to as pleiotropic if it has associations with more than one risk factor on different causal pathways. Any such effect is included in$\alpha_{j}$, a genetic variant is pleiotropic if $\alpha_{j}\neq0$. A pleiotropic genetic variant violates the IV3 assumption, and is not a valid instrumental variable [1].

For a given genetic variant that meets the instrumental variable assumptions ($\alpha_{j}=0$), the causal effect of the risk factor on the outcome can be consistently estimated as a simple ratio of association estimates:

$\theta^{\wedge} _{j}=\frac{\beta^{\wedge} _{Yj}}{\beta^{\wedge} _{Xj}}$, their approximate variances

**Inverse-variance weighted method**

When there are multiple genetic variants, the inverse-variance weighted (IVW) estimate is the weighted average of these causal estimates, using the inverse of their approximate variances as weights:

This estimate can be estimated using a weighted linear regression of the genetic associations with the outcome ($\beta^{\wedge} _{Yj}$) on the genetic associations with the risk factor ($\beta^{\wedge} _{Xj}$) using inverse variance weights ($se(\beta^{\wedge} _{Yj})^{-2}$) when the intercept is zero:

$\beta^{\wedge} _{Y_{j}}=\theta_{UI}\beta^{\wedge} _{Xj}+\varepsilon_{UI_{j}}$, weights=$se(\beta^{\wedge} _{Yj})^{-2}$

Where $\varepsilon_{UI_{j}}$is the residual term. If the residual standard error is set to one, this above weighted regression model, is equivalent to performing a fixed-effect meta-analysis [1]. If the pleiotropic effects of the genetic variants are all zero ($\alpha_{j}=0$ ($j=1,...,J$), in other words, if all genetic variants are valid instrumental variables), then each of the $\theta^{\wedge} _{j}$ will be a consistent estimate of the causal effect, and the overall estimate $\theta^{\wedge} _{UI}$ (a weighted mean of the $\theta^{\wedge} _{j}$) will be a consistent estimate of the causal effect [1].

**Reference**

1. Burgess S, Thompson SG: **Interpreting findings from Mendelian randomization using the MR-Egger method**. *Eur J Epidemiol* 2017, **32**(5):377-389.
